# Prediction and trend of tactile acuity, pain and disability in acute LBP: a six-month prospective cohort study

**DOI:** 10.1186/s12891-021-04530-z

**Published:** 2021-08-09

**Authors:** Rita Morf, Fabian Pfeiffer, Sabina Hotz-Boendermaker, André Meichtry, Hannu Luomajoki

**Affiliations:** 1 Zurich University of Applied Sciences (ZHAW), School of Health Professions, Institute of Physiotherapy, Katharina-Sulzer-Platz 9, Postfach, CH–8401 Winterthur, Switzerland; 2 Physiotherapy Medbase Winterthur, Archplatz 4, CH-8400 Winterthur, Switzerland; 3 Zurich University of Applied Sciences, School of Health Professions, Institute of Physiotherapy, Katharina-Sulzer-Platz 9, CH-8400 Winterthur, Switzerland

**Keywords:** Tactile acuity, Pain, Disability, LBP

## Abstract

**Background:**

Chronic back pain is known to be associated with altered tactile acuity. Tactile acuity is measured using the Two-Point Discrimination (TPD) test in both clinical and research settings. In subjects with chronic low back pain, the TPD threshold (TPDT) is increased and is associated with persistent pain. It remains unknown, however, whether TPDT is also altered in cases of clinical acute pain, or whether it could be used as a predictor of future pain and disability at an early stage of LBP.

The main objective of this study was to investigate the predictive value of baseline TPDT for pain and disability at 3 and 6 months after the onset of acute LBP. The TPDT in acute low back pain (LBP) and the development of TPDT over 6 months has also been assessed.

**Methods:**

LBP participants (*n* = 124) with acute LBP (< 4 weeks) were included. Subjects were examined within 4 weeks of pain onset and followed-up after 3 and 6 months of pain onset. Horizontal and vertical TPDTs of the lower back were collected. Linear mixed models were subsequently used to evaluate the association of TPDT with pain and disability over time.

**Results:**

The vertical TPDT showed a mean (SD) of 4.9 cm (1.6) and the horizontal TPDT a mean (SD) of 6.0 cm (1.5) at baseline. The vertical TPDT altered from baseline up to 6 months from 4.9 to 4.6 cm and the horizontal TPDT from 6.0 to 5.4 cm. The association between the TPDT and the Oswestry Disability Index (ODI) after 6 months was moderate. Linear mixed models revealed no association between TPDT, pain and disability over the progression of LBP.

**Conclusion:**

TPDTs appear to be raised in subjects with acute LBP. However, our study revealed no predictive capability of the TPDT for disability and pain. No comparisons are possible in the absence of similar studies, indicating the need for further research is in this area.

## Background

With a lifetime prevalence of up to 85% [1], low back pain (LBP) is the most common symptom of all musculoskeletal disorders [2]. Within the first 2 months of the onset of pain, most subjects show substantial improvements in pain and disability [2]. However, within 1 year after recovery from an acute episode of LBP, 69% of subjects suffer recurrent LBP [3]. On average, about 3–10% of individuals develop persistent pain after an acute episode of LBP. In addition, these individuals do not return to work afterwards [4]. This transition from acute LBP to chronic LBP (CLBP) is not linear [4]. In general, CLBP was defined as persistent or recurrent pain lasting longer than 3 months and is associated with emotional stress and/or significant functional disability [5]. Besides, 15% of subjects diagnosed with CLBP show no improvement after 2 years [6]. CLBP can lead to substantial health-related costs and is responsible for an increasing socio-economic burden [7, 8]. Dynamic maladaptive interactions between physiological, psychological and social factors increase the likelihood of chronic pain and disability [9]. Pain intensity, duration resp. frequency, and coping strategies are important predictors of chronic pain itself [10–12]. In addition, baseline values of depression and maladaptive cognitions are clinical predictors of pain intensity and disability after 6 months [13]. These results indicate the necessity to identify high-risk LBP subjects at the earliest possible stage [14, 15]. To date, besides psychosocial variables, few physical examinations have been shown to be predictive of pain persistence. To close this gap, we therefore propose tactile acuity as a novel prospective assessment tool.

### Tactile acuity

Tactile acuity is described as the perceived precision of touch [16] and has been found to be decreased in various chronic pain conditions [17]. Moreover, tactile acuity is thought to represent a simple clinical measure of a cortical representation of tactile perception [18]. It can be measured by means of two-point discrimination (TPD). TPD is defined as the ability to perceive the smallest distance between two tactile stimuli, placed at distinct points on the skin [19]. Tactile acuity is reduced in subjects with CLBP [20, 21], resulting in higher TPDTs compared with healthy subjects [22]. Cross-sectional data have revealed no significant differences in TPDT between the affected and non-affected sites in unilateral CLBP [23]. In addition, vertical TPDTs are usually lower in comparison to horizontal TPDTs [20]. The extent to which the TPDT is affected in subjects at the acute stage of clinical LBP is still unknown. Similarly, the predictive value of the TPDT for the development of CLBP has not yet been investigated.

The main objective of this study is to investigate TPDTs in acute LBP and follow-up their longitudinal course over a 6-month period, with the aim to assess the predictive value of the TPDTs for pain and disability.

## Methods

### Study design

This project was part of a larger prospective longitudinal cohort study. The overall study examines the setting, physical factors and psychological factors of LBP subjects, with a follow-up period of up to 1 year. In this repeated measure design study, subjects were investigated within the first 4 weeks of the onset of acute LBP (T1), at 3 months (T2), and finally at 6 months (T3). This part of the study focused on the measures of tactile acuity, pain and disability and the associations between them.

The study protocol is in accordance with the Declaration of Helsinki and approval was obtained from the Ethics Committee of the Canton of Zurich (BASEC-No. 2016–02,096). All experiments were performed in accordance with relevant guidelines and regulations.

### Subjects

Subjects aged 18–65 years suffered from acute LBP. Inclusion criteria required them to have been pain-free for a 3-month period prior to the onset of the current episode. Access to the internet and a good knowledge of the German language were further inclusion criteria. Excluded were persons who showed signs of serious pathologies, had given birth within the previous 12 months, were currently pregnant, had a history of severe psychiatric disorder, used psychiatric medications, or had progressive neurological symptoms.

### Recruitment

Subjects were recruited in hospitals, private physiotherapy practices and a university campus in the canton of Zurich (Switzerland). They were either contacted personally, via the university campus homepage, intranet, flyers, advertisements or per email. The selection criteria were reviewed prior to the first examination and signed informed consent was obtained.

### Data collection

Various experienced physiotherapists carried out the clinical tests. To standardise the test procedures, the assessors received a manual with instructions for all tests and were trained in advance. Because the intra-rater reliability of TPDT measurement has been shown to be high in healthy individuals, the measurements were performed by the same assessor whenever possible [24]. They were also blinded to the initial screening and to the results of the psychometric assessments of the subjects.

### Measurements

For both TPDT measurements a horizontal line at the level of L3 was used as reference. The vertical TPDT was measured between Th12 and the S1 above the erector muscle on each side of the lumbar spine with the starting point on the transversal line of L3.The horizontal TPDT was measured on the transversal line of L3 and with 5 cm between the tips of the plastic caliper and the lumbar spine in closed position. The stimulation intensity was defined as ‘the slight touch of the skin on the back until the occurrence of the first blanching’ [24, 25]. TPDT was measured in 5 mm increments between 1–10 cm, one run ascending and one run descending. The procedure was ended as soon as the subject stated that he felt two points in the ascending measurement and one point in the descending measurement. Subjects were invited to verbally express the number of perceived touches on the skin. Average values of the descending and ascending values were then calculated. In healthy adults, a mean TPDT value of 55.5 mm (12.5) has been determined [24], whereas TPDTs have been shown to be wider in subjects with CLBP [22].

Pain intensity was measured using the Numeric Rating Scale (NRS). The NRS is a single 11-point numeric scale ranging from 0 to 10, with 0 representing “no pain” and 10 representing “worst pain you can imagine” [26].

Disability was assessed using the German version of the Oswestry Disability Index (ODI-D). The self-administered questionnaire assesses functional status, with substantive reliability (*r* = 0.96) and construct validity (*r* = 0.80) [27]. The Oswestry Disability Index (ODI) score is applied as follows: 0–20% = minimal disability; score ≤ 21- 40% = moderate disability; score ≤ 41- 60% = severe disability; score ≤ 61–80% = crippling disability; ≤ 80–100% = bed-bound [28]. It has been found useful for monitoring subjects in clinical practice and as an outcome measure for clinical trials [27, 29].

The ODI questionnaire was completed by subjects online. It was required to be completed within 2 days of the date of request. The invitations were sent to subjects by email by the study director. If a respondent did not complete a questionnaire within the required time, an electronic reminder was sent. This was then followed by a telephone call if they had not responded to the request.

### Data analysis

A subject was defined as a drop-out where there was: missing data in two subsequent measurements; an unreliable answer of more than one-week delay; or, withdrawal from the study. In our regression analysis, a list-wise deletion was performed to remove the series of values for which an observation was missing. Subsequently, the maximum likelihood was used to obtain estimates of the model parameters.

Descriptive statistics and a spearmen rank correlation analysis were initially applied to screen for disproportional subject characteristics, data outliers and absences of collinearity. Spearman rank correlations were categorized using the interpretation table [30]. Following this, linear mixed regression models were fitted to the data to evaluate the effect of the independent variables on disability and pain over time. Age was included as a potential confounding variable based on its association with the TPDT [31]. Timepoints (T2 and T3) were entered as fixed effects and subjects as random effects (intercepts). The following equation describes the model:

$${Y}_{i,j}={\upbeta }_{0}+{\upbeta }_{\mathrm{1,1}}I\left(tim{e}_{i,j}=T2\right)+{\upbeta }_{\mathrm{1,2}}I\left(tim{e}_{i,j}=T3\right)+{\upbeta }_{2}TPD{T}_{i,j}+{\upbeta }_{\mathrm{3,1}}TPD{T}_{i,j}I\left(tim{e}_{i,j}=T2\right)+{\upbeta }_{\mathrm{3,2}}TPD{T}_{i,j}I\left(tim{e}_{i,j}=T3\right)+C+{U}_{i}+{\upepsilon }_{i,j}$$

with ${Y}_{i,j}$ representing ODI or pain intensity for subject $i$ at time point $j$=T1, T2, T3.

${\upbeta }_{0}$ represents the intercept, $I$ the indicator function, ${\upbeta }_{\mathrm{1,1}}$ and ${\upbeta }_{\mathrm{1,2}}$ the time effect of time T2 and T2, respectively, ${\upbeta }_{2}$ the effect of TPDT, ${\upbeta }_{\mathrm{3,1}}$ and ${\upbeta }_{\mathrm{3,2}}$ the interaction effect at time T2 and T2, respectively, $C$ the effect of the confounding factor (in this case, age), ${U}_{i}$ the random effect (in this case, subjects) ${\upepsilon }_{i,j}$ the error term. The individual mean for the vertical and horizontal TPDT was calculated including the right and left side values. Four different models were fitted to the data: 1. Baseline horizontal TPDT as predictor and pain intensity over time as dependent variable; 2. Baseline horizontal TPDT as predictor and ODI over time; 3. Baseline vertical TPDT as predictor and pain intensity over time; 4. Baseline vertical TPDT as predictor and ODI over time.

The vertical and horizontal TPDTs were evaluated individually against the outcomes, since it is known that these measurements yield different values [20]. The effect on pain intensity and ODI was analysed based on the given clinically minimal important change [32]. All analyses were performed using the R statistical software R version 3.6.3 (2020–02-29).

Incomplete measurement data from patients who did not complete all 3 measurements were excluded from the evaluation of the linear mixed model.

## Results

### Characteristics of subjects

A total of 124 subjects were recruited in the period from November 2017 to December 2019. Of these subjects, 21 dropped out for the following reasons: time constraints (6); health issues (pregnancy 2, back surgery 1, spine fracture 1, no precise information (3)); personal reasons (1); respondent not adhering to specifications (2); dissatisfaction with the scheduling (1); no information (1); no response to contact (3). On average, the subjects were 41 years old (SD 12.7) and 49 subjects were female (48%). Table 1 illustrates the characteristics of the included subjects.

**Table 1:** Subject Characteristics at time point T1 (*N* = 124)

	**Baseline Values T1**
**Sex**	
Female	49 (47.6%)
Male	51 (49.5%)
No data	3 (2.9%)
**Age (years)**	
Range	21–65
Mean (SD)	41 (12.7)
**Pain Intensity**	
Pain intensity (NRS, range = 0–10), mean (SD)	2.5 (2.1)
NRS < 3	64%
NRS > 3	34%
No data	2%
**Disability**	
ODI (0–100%), mean (SD)	37 (11.2)
Minimal disability	5%
Moderate disability	62%
Severe disability	23%
Crippling disability	5%
No data	5%
**TPDT**	
TPDT vertical (1–10 cm), mean (SD)	4.9 (1.6)
TPDT horizontal (1–10 cm), mean (SD)	6.0 (1.5)

*Pain intensity* Numeric Rating Scale (NRS) 0–10, *Disability* Oswestry Disability Index (ODI): score ≤ 20% = minimal disability; score 21- 40% = moderate disability; score ≤ 41- 60% = severe disability; score > 61–80% = crippling disability; score > 81% bed-bound [28], *TPDT* Two-point discrimination threshold measured with a plastic calliper ruler in vertical and horizontal direction from 1–10 cm

### TPDT in the acute pain state

At baseline, the mean TPDTs measured in this study were as follows: mean (SD) 4.9 cm (1.6) in the vertical direction and 6.0 cm (1.5) in the horizontal direction.

### Time progression of the TPDT, ODI and pain intensity

**Fig. 1:**
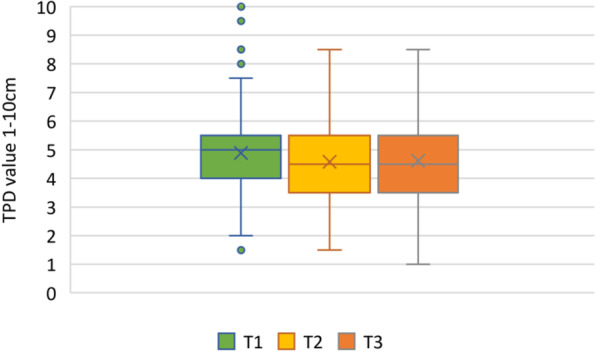
Time course of the vertical TPDTs over 6 months. T1: timepoint 1 (< 4 weeks), T2: timepoint 2 (3 months), T3: timepoint 3 (6 months). TPDT: Two-point discrimination threshold measured with a plastic calliper ruler in vertical and horizontal direction from 1-10 cm

**Fig. 2:**
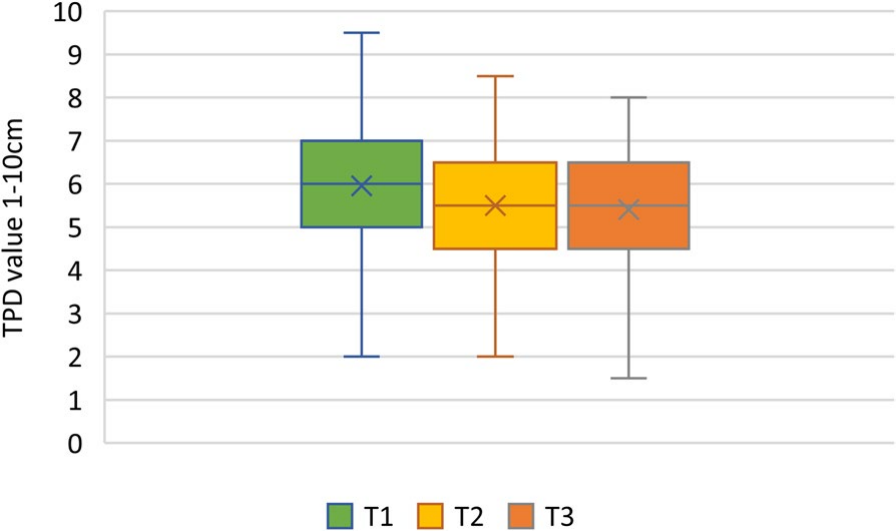
Time course of the horizontal TPDTs over 6 months. T1: timepoint 1 (< 4 weeks), T2: timepoint 2 (3 months), T3: timepoint 3 (6 months). TPDT: Two-point discrimination threshold measured with a plastic calliper ruler in vertical and horizontal direction from 1-10 cm

**Fig. 3:**
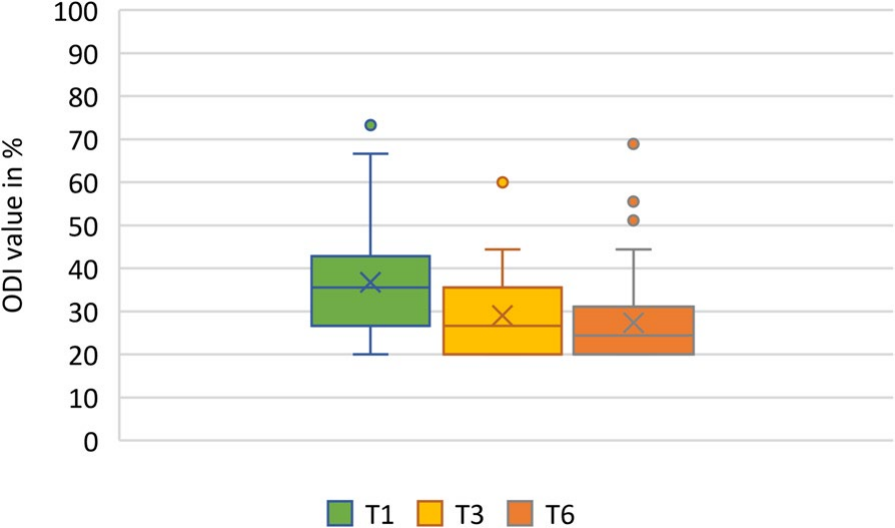
Time course of the ODI over 6 months. T1: timepoint 1 (< 4 weeks), T2: timepoint 2 (3 months), T3: timepoint 3 (6 months). ODI: Oswestry Disability Index (ODI-Index): Score ≤ 20% = minimal disability; Score 21- 40% = moderate disability; Score ≤ 41- 60% = high disability; Score 61–80% = very high disability; Score > 81% in need of care or psycho-socially extremely overlaid (28). NRS: Numeric Rating Scale (NRS) 0–10

**Fig. 4:**
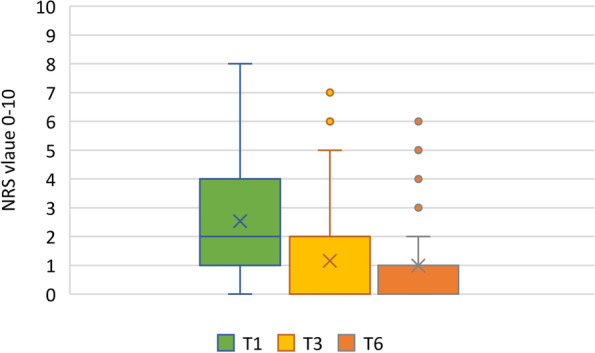
Time course of the NRS over 6 months. T1: timepoint 1 (< 4 weeks), T2: timepoint 2 (3 months), T3: timepoint 3 (6 months). ODI: Oswestry Disability Index (ODI-Index): Score ≤ 20% = minimal disability; Score 21- 40% = moderate disability; Score ≤ 41- 60% = high disability; Score 61–80% = very high disability; Score > 81% in need of care or psycho-socially extremely overlaid [28]. NRS: Numeric Rating Scale (NRS) 0–10

The mean value of the vertical TPDT changed over 6 months from T1: 4.9 cm to T2: 4.6 cm to T3: 4.6 cm. The horizontal TPDT mean value altered from T1: 6.0 cm to T2: 5.5 cm to T3: 5.4 cm. For disability, the mean ODI index decreased over 6 months from T1: 37 to T2: 29 to T3: 27. The pain intensity mean value decreased from T1: 2.5 to T2: 1.16 to T3: 0.99 over 6 months. Figures 1, 2, 3 and 4 illustrate the time progression of the variables with box plots. Table 2 shows in-depth information on the response variables ODI and pain intensity, as well as on the predictor TPDT.

**Table 2:** Time progression of the variables

	**T1**		**T2**		**T3**	
	Subjects	Frequency	Subjects	Frequency	Subjects	Frequency
**Vertical TPDT**						
≥ 6 cm	21	20%	15	15%	15	15%
< 6 cm	82	80%	67	65%	59	57%
No data	0	0%	21	20%	29	28%
**Horizontal TPDT**						
≥ 6 cm	59	57%	37	36%	31	30%
< 6 cm	44	43%	45	44%	43	42%
No data	0	0%	21	20%	29	28%
**ODI**						
Minimal disability	5	5%	22	21%	29	28%
Moderate disability	64	62%	52	50%	40	39%
Severe disability	24	23%	9	9%	6	6%
Crippling disability	5	5%	2	2%	1	1%
No data	5	5%	18	17%	27	26%
**NRS**						
NRS 0	-	-	46	45%	43	42%
NRS 1–3	66	64%	27	26%	23	22%
NRS ≥4	35	34%	9	9%	9	9%
No data	2	2%	21	20%	28	27%

*T1* Time point 1 (< 4 weeks), *T2* Time point 2 (3 months), *T3* Time point 3 (6 months), *TPDT* Two-point discrimination threshold measured with a plastic calliper ruler in vertical and horizontal direction from 1–10 cm, *ODI* Oswestry Disability Index score ≤ 20% = minimal disability; score 21- 40% = moderate disability; score ≤ 41- 60% = severe disability; score 61–80% = crippling disability; score > 81% bed-bound [28], *NRS* Numeric Rating Scale (NRS) 0–10

Spearman rank correlation analysis showed fair correlations between the vertical TPDTs, the ODI and pain intensity at T3. Weak correlations were observed at T1 and weak negative correlations at T2. Fair correlations with the ODI were also found for the horizontal TPDTs at T2 and at T3. A weak correlation was observed at T1. In the case of horizontal TPDTs and pain intensity in T1 positive weak correlations were detected and T2-T3 showed negative weak correlations. Large confidence intervals could be detected in almost all calculations. Table 3 shows the Spearman Rank correlations and confidence intervals of TPDT and ODI/pain intensity.

**Table 3:** Spearman Rank Correlations of TPDT and ODI/NRS

	**N**	**R**	**95% CI**
**TPDT vertical / ODI**			
T1	98	0.12	–0.08 to 0.31
T2	82	–0.05	–0.25 to 0.16
T3	74	**0.33**	0.12 to 0.53
**TPDT vertical / NRS**			
T1	101	–0.06	–0.25 to 0.14
T2	82	0.06	–0.17 to 0.26
T3	74	**0.25**	0.01 to 0.46
**TPDT horizontal / ODI**			
T1	98	0.15	–0.08 to 0.35
T2	82	**0.27**	0.03 to 0.49
T3	74	**0.38**	0.15 to 0.58
**TPDT horizontal / NRS**			
T1	101	0.03	–0.17 to 0.23
T2	82	–0.14	–0.35 to 0.06
T3	74	–0.03	–0.26 to 0.21

*N* Number of subjects, *R* Spearman Rank Correlation, *95%CI* 95% confidence interval, *NRS* Numeric Rating Scale (NRS) 0–10, *ODI* Oswestry Disability Index score 0–100%, *TPDT* Two-point discrimination threshold in vertical and horizontal direction from 1–10 cm

### Predictive value of baseline TPDT

Our analysis evaluated the interaction effects with time of baseline vertical and horizontal TPDTs on disability and pain over the 6-month measurement period. Baseline TPDTs had no significant effects on either ODI or pain intensity (Tables 4, 5, 6 and 7). Furthermore, the ODI decreased over time, which was found in both the horizontal and vertical TPDT evaluations. Similar effects were found for pain intensity, which also decreased over time. The primary analysis showed no relevant time-predictor interaction effects on ODI and pain intensity. In the evaluations with ODI, negative time-predictor interaction effects were found with the TPDT for vertical and horizontal TPDT between both T1 / T2 and T1 / T3. In the evaluations with pain intensity similar negative time-predictor interaction effects were found with the TPDT for vertical and horizontal TPDT but only between T1 / T2.

**Table 4:** Linear mixed model for ODI with vertical TPDT as predictor

**Parameter**	**Estimate**	**SE**	**95% CI**
Intercept, β_0_	29.47	4.18	21.34 to 37.59
Vertical TPDT, β_2_	–0.44	0.67	–1.73 to 0.86
T2, β_1,1_	–2.95	3.46	–9.74 to 3.77
T3, β_1,2_	–5.20	3.61	–12.32 to 1.80
Age, C	0.23	0.08	0.09 to 0.38
Vertical TPDT x T2, β_3,1_	–0.86	0.66	–2.15 to 0.44
Vertical TPDT x T3, β_3,2_	–0.83	0.69	–2.18 to 0.53

*TPDT* Two-point discrimination threshold, *ODI* Oswestry Disability Index, *T1* Time point 1 (< 4 weeks), *T2* Time point 2 (3 months), *T3* Time point 3 (6 months), *Vertical TPDT T1xT2* and *Vertical TPDT T1xT3* Interaction effects of TPDT and time on ODI, *Estimate* Estimated fixed effects, *SE* Standard Error, *95% CI* 95% confidence interval of estimated effect

**Table 5:** Linear mixed model for ODI with horizontal TPDT as predictor

**Parameter**	**Estimate**	**SE**	**95% CI**
Intercept, β_0_	28.50	4.68	19.41 to 37.59
Horizontal TPDT, β_2_	–0.14	0.70	–1.50 to 1.22
T2, β_1,1_	–1.89	4.27	–10.20 to 6.43
T3, β_1,2_	–5.78	4.44	–14.46 to 2.86
Age, C	0.23	0.08	0.08 to 0.37
Horizontal TPDT x T2, β_3,1_	–0.90	0.69	–2.25 to 0.45
Horizontal TPDT x T3, β_3,2_	–0.60	0.73	–2.02 to 0.81

*TPDT* Two-point discrimination threshold, *ODI* Oswestry Disability Index, *T1* Time point 1 (< 4 weeks), *T2* Time point 2 (3 months), *T3* Time point 3 (6 months), *Horizontal TPDT T1xT2* and *Horizontal TPDT T1xT3* Interaction effects of TPDT and time on ODI, *Estimate* Estimated fixed effects, *SE* Standard Error, *95% CI* 95% confidence interval of estimated effect

**Table 6:** Linear mixed model for NRS with vertical TPDT as predictor

**Parameter**	**Estimate**	**SE**	**95% CI**
Intercept, β_0_	1.97	0.69	0.62 to 3.31
Vertical TPDT, β_2_	–0.08	0.12	–0.30 to 0.15
T2, β_1,1_	–0.88	0.77	–2.38 to 0.61
T3, β_1,2_	–1.59	0.78	–3.11 to –0.08
Age, C	0.02	0.01	–0.00 to 0.04
Vertical TPDT x T2, β_3,1_	–0.08	0.15	–0.36 to 0.21
Vertical TPDT x T3, β_3,2_	0.04	0.15	–0.25 to 0.32

*TPDT* Two-point discrimination threshold, *NRS* Numeric Rating Scale, *T1* Time point 1 (< 4 weeks), *T2* Time point 2 (3 months), *T3* Time point 3 (6 months), *Vertical TPDT T1xT2* and *Vertical TPDT T1xT3* Interaction effects of TPDT and time on NRS, *Estimate* Estimated fixed effects, *SE* Standard Error, *95% CI* 95% confidence interval of estimated effect

**Table 7:** Linear mixed model for NRS with horizontal TPDT as predictor

**Parameter**	**Estimate**	**SE**	**95% CI**
Intercept, β_0_	1.58	0.79	0.05 to 3.12
Horizontal TPDT, β_2_	0.03	0.12	–0.21 to 0.26
T2, β_1,1_	–1.02	0.95	–2.87 to 0.82
T3, β_1,2_	–1.85	1.00	–3.81 to 0.10
Age, C	0.02	0.01	0.0 to 0.04
Horizontal TPDT x T2, β_3,1_	–0.04	0.15	–0.34 to 0.26
Horizontal TPDT x T3, β_3,2_	0.07	0.16	–0.25 to 0.39

*TPDT* Two-point discrimination threshold, *NRS* Numeric Rating Scale, *T1* Time point 1 (< 4 weeks), *T2* Time point 2 (3 months), *T3* Time point 3 (6 months), *Horizontal TPDT T1xT2* and *Horizontal TPDT T1xT3* Interaction effects of TPDT and time on NRS, *Estimate* Estimated fixed effects, *SE* Standard Error, *95% CI* 95% confidence interval of estimated effect

## Discussion

Our data show horizontal TPDTs ≥ 6 cm in acute LBP patients and slightly decreasing TPDTs over 6 months. Furthermore, moderate correlations were found between the TPDT, the ODI and pain intensity. Our study provides no evidence that baseline values of TPDTs are predictors of persistent pain or disability in CLBP.

### TPDT in the acute pain state

While there is a well-established body of literature on the TPDT for healthy adults, there is no comparable work on the TPDT for clinical acute LBP subjects. Healthy volunteers without back pain showed TPDTs as follows: TPDT mean (SD) vertical left 4.32 cm (1.58), vertical right 4.33 cm (1.44), horizontal left 4.53 cm (1.13) and horizontal right 4.46 cm (1.14) in 25–61-year olds [20]. Current findings show that TPDTs are higher in subjects with CLBP compared to healthy subjects [21, 22]. Our study in subjects with clinical acute LBP shows higher TPDTs: mean (SD) 4.9 cm (1.6) in the vertical direction and 6.0 cm (1.5) in the horizontal direction. In addition, TPDT is also increased in experimental acute LBP with deterioration of TPDT mean (SD) from 5.7 cm (0.7) to 6.4 cm (0.8) shortly after pain was induced [33]. These comparable results support the current understanding of altered TPDT in an acute pain state. Thus, it is possible that TPDTs are generally elevated in subjects with LBP regardless of the duration of LBP. The reason for increased TPDT in subjects with acute experimental LBP was thought to be the nociceptive pain itself [33]. However, in other pain states it remains unclear and requires further investigation.

The observation of the baseline TPDTs in this study showed larger TPDTs in horizontal direction compared to vertical direction. This finding is in line with the results of others [20], which also found higher horizontal TPDTs although in healthy volunteers. Movement in the frontal direction might stretch the skin in the vertical direction, thus smaller TPDT would hamper the adequate skin response [20].

Standardised TPDT assessment procedures do not yet exist. This affects the interpretation and comparability of study results. Amongst other reasons, the stimulus size has shown to be an important factor in TPDT assessments [34–36].

Earlier studies reported on the use of pressure until the very first blanching of the skin [24, 37], whereas other assessment procedures use 1 mm skin pressure to standardise stimulus levels [38]. In addition, the TPDT protocol [35], measurement instruments [39] and intra-rater capabilities [40] contribute to between and within subject variability. A standardised TPDT measurement would certainly enhance the interpretation of different results. In addition, inherent natural variability of tactile acuity in subjects with LBP and healthy controls might contribute to the variability of TPDT. It is likely that some of these factors contributed to the variability observed in this study.

### Time progression of the TPDT, ODI and pain intensity

Our findings show that TPDTs change only minimally over a period of 6 months. This indicates that the TPDTs remain unchanged without further treatment. Previous studies on subjects with CLBP have demonstrated that sensory discrimination training can improve pain and function [41]. Thirty minutes of tactile acuity training for CLBP subjects is sufficient to achieve an improvement in the TPDT [42]. However, whether subjects with acute LBP would also benefit from tactile acuity training remains unclear. Given the fact that chronic and acute LBP revealed similar TPDTs, tactile acuity training might work in a similar way.

In terms of disability, this study shows a substantial decrease for the ODI index over 6 months, as shown previously.

Regarding pain, the study findings demonstrate a steep decrease in the pain intensity, especially within the first 3 months and a lower decrease of the NRS value after 6 months. Similar observations have been reported previously investigated in acute LBP up to 3 and 6 months [43].

Some 35% of the test persons in the study still suffered from pain after 3 months, with the rate remaining at about 31% at the end of the 6 months. By definition, about one third of the subjects therefore suffered from chronic pain, since a patient is considered cured only when the cut-off NRS 0/10 is not exceeded [44].

However, one should be careful to assume that the progression of pain and disability over 3 and 6 months is the same for each person. From other studies, we are aware that the progression for an individual subject can be completely different from the mean group progression [45]. Furthermore, LBP is not a condition in which rapid recovery is experienced or chronic severe pain developed. In contrast, LBP might be interpreted as a state of persistent or fluctuating pain of low or moderate intensity [46].

### Prediction of pain and disability

The regression analysis showed no predictive value of the TPDT for disability or pain at 3 and 6 months after pain onset. To the best of our knowledge, this is a novel finding and has not been demonstrated so far.

The results of the regression analysis with the ODI were puzzling. The correlation between the ODI and the TPDT was negative at all time points (T1-T3). In the evaluations with pain intensity, similar negative interaction effects were found with the TPDT at T2. Moderate correlations were found between the TPDT and the ODI after 6 months. There were only weak correlations between the TPDT and the pain intensity. These results agree with findings from other studies, demonstrating that tactile acuity deficits may be independent of the perceived intensity of pain [22].

The overall large confidence intervals of the estimated effects demonstrate the difficulty in generalising our results and shows that there is a wide spread of values and thus the conclusion of the correlations become more uncertain.

It may be concluded that TPDT, NRS and ODI values do not behave similarly because they measure different constructs. While the TPDT is a measurement of skin perception, the NRS measures pain intensity as a subjective sensory experience and the ODI index assesses patient subjective abilities in daily tasks. A comparison of these measures may therefore not be meaningful.

### Strengths and limitations

This study is the first prospective longitudinal study to investigate the ability of the TPDT to predict pain and disability. The high dropout rate of 16.9% over the period of 6 months led to a certain loss of data and must be considered when interpreting the results. Furthermore, this study was embedded in a larger project, in which a huge amount of additional data was collected. Adherence to the defined examination dates also led to a high burden on the test subjects.

The generalisability of the results is weakened by the lack of a representative population sampling. Due to the localisation of recruitment, many young and well-educated subjects were included. Additionally, the TPDT measurement could not always be performed by the same test person, due to the large number of assessors and to the fact that they were part-time students. Furthermore, the absence of repeated measurements at baseline must be considered as a potential confounder for the interpretation of the results.

More baseline data regarding "chronicity risk" would have allowed for more complex ways of looking at the process. Stratification of participants according to their risk of future disability would have been useful to identify participants most likely to be affected by a future decline in tactile acuity [47].

## Conclusion

This study investigated the ability of TPDTs to predict pain and disability in acute LBP subjects over a period of 6 months, using measurements of vertical and horizontal TPDTs at 3 and 6 months. The study demonstrated elevated TPDTs in acute LBP persons and only minimal changes in TPDTs over the 6-month period. The results indicate that TPDT has no predictive value for disability and pain at 3 and 6 months after pain onset. Therefore, further research is needed to clarify the effects and therapeutic value of TPDT in acute LPB.

## Data Availability

The data sets used and analysed in the current study are available on request from the corresponding author.

## Abbreviations

CLBP: Chronic low back pain
LBP: Low back pain
NRS: Numeric Rating Scale
ODI: Oswestry Disability Index
TPD: Two-point discrimination
TPDT: Two-point discrimination threshold
Th12-S1: Region in the lower back from 12. thoracic vertebrae to 1. lumbar vertebra
